# Geometric response of curved mandibular molars to NiTi instrumentation

**DOI:** 10.6026/973206300221881

**Published:** 2026-03-31

**Authors:** Juveria Masreen, Vijetha Badami, Bhavana Badaka, Shaik Mohammad Waseem, Alla Sriharsha, Yoshna Sree V

**Affiliations:** 1 Department of Conservative Dentistry and Endodontics, MNR Dental College and Hospital, Sangareddy, Telanagana, India

**Keywords:** Nickel-Titanium rotary systems, micro-computed tomography (micro- CT), root canal transportation, centering ability, hyflex EDM, trunatomy, XP endo rise, mandibular first molars

## Abstract

Preserving root canal geometry in curved canals remains a major challenge during mechanized NiTi instrumentation. Therefore, it is of interest to evaluate the shaping performance of HyFlex EDM, TruNatomy and XP-Endo Rise in curved mesiobuccal canals using high-resolution micro-computed tomography. Thirty extracted mandibular first molars with 15°C-35°C curvature were allocated into three groups (n = 10) and canal transportation and centering ability were assessed at 3, 6 and 9 mm from the apex. XP-Endo Rise showed minimal buccolingual but increased mesiodistal transportation, whereas TruNatomy demonstrated superior centering with minimal canal deviation and HyFlex EDM exhibited balanced shaping behavior. All systems maintained root canal geometry within clinically acceptable limits.

## Background:

Effective biomechanical preparation in conjunction with maintaining the root canal systems inherent anatomy is critical for endodontic success [1]. Root canals with marked curvature, especially mesial canals of mandibular first molars, remain challenging to instrument and are highly susceptible to transportation errors [2]. The prevalence of canal curvature in mandibular molars ranges from 3.3% to 30.9%, with 47.4% reported in these teeth [3]. Their anatomical complexity, frequently characterized by Vertucci Type II or IV configurations and proximity to the distal concavity ("danger zone"), demands meticulous preservation of canal curvature [4]. Canal transportation, defined by the American Association of Endodontists as the removal of dentin from the outer curve of the apical canal, typically results from a file's tendency to straighten and can lead to ledging, perforation, or compromised tooth structure [5]. The introduction of nickel-titanium (NiTi) rotary systems have greatly optimized root canal shaping by enhancing flexibility and reducing errors [6]. Advances in metallurgy (*e.g.*, M-Wire, Controlled Memory wire) and manufacturing (electrical discharge machining) have improved cyclic fatigue resistance and centering ability [7]. Modern designs, including non-cutting tips, variable tapers and off-centered cross-sections, further improve safety and efficiency [8]. This study employs micro- computed tomography (micro-CT) to conduct a comparative analysis of the shaping ability of Trunatomy, XP-endo Rise and Hyflex EDM in curved mesial canals of extracted mandibular first molars. The null hypothesis stated that there would be no significant difference in canal transportation and centering ability among these systems. Though modern NiTi systems show promise, scarce comparative data and rapid innovations demand objective, standardized evaluation for informed clinical decisions. Therefore, it is of interest to investigate the shaping characteristics of three contemporary NiTi systems using micro-CT analysis.

## Materials and Methods:

This *in vitro* study was conducted at the Department of Conservative Dentistry and Endodontics, MNR Dental College and Hospital, Sangareddy, in collaboration with the Indian Institute of Technology, Hyderabad. The study protocol was approved by the Institutional Ethics Committee (IEC No. MNRDCH/IEC/2022-23/01). Forty human mandibular first molars, extracted for periodontal reasons, were obtained from the Department of Oral and Maxillofacial Surgery, MNR Dental College and stored in 10% buffered formalin until use. Based on prior literature, thirty extracted teeth constituted the study sample. Following ultrasonic scaling, the specimens were sterilized in an autoclave at 120°C and 20 psi for 20 minutes. To prevent dehydration, throughout the experimental period, specimens were kept in isotonic saline at room temperature. Thirty samples were selected that satisfied the inclusion and exclusion criteria. Mesiobuccal roots with a confirmed Vertucci Type IV mesial canal configuration were exclusively included in the study. Included canals showed 15°C-35°C curvature by Schneider's method on standardized RVG images and an initial apical size of #10 K-file. Teeth with incomplete root formation, open apices, resorption, root caries, fractures, severe canal curvature (>35°C), or apical diameters larger than a #10 K-file were excluded. Using diamond discs, the distal halves were resected and the mesial roots were decoronated 2 mm above the cementoenamel junction (CEJ). A customized plywood jig was used to standardize radiographic positioning [9], as shown in (Figure 1 - see PDF), Buccolingual radiographs were taken using the Carestream Kodak RVG 5200 digital sensor (65 kVp, 8 mA, 0.6 s exposure) to confirm eligibility. Canal curvature and radius were assessed using Schneider's method via the EndoPrep application. Thirty mesiobuccal canals were randomly categorized into three experimental groups (n = 10 each) using stratified random sampling. Owing to the distinct designs of the instruments, operator blinding was not feasible. A blinded second examiner conducted the micro-CT analysis. Working length was established by advancing a size #10 K-file to the point of apical foramen visualization and subtracting 0.5 mm. All canals were instrumented by a single trained operator using an X-Smart plus endodontic motor (Dentsply Maillefer). Irrigation with 2ml of 3% sodium hypochlorite was carried out between each instrument use, followed by a rinse with 5ml of 17% liquid ethylenediaminetetraacetic acid (EDTA) for one minute and a final flush with 5ml normal saline.

All specimens were scanned twice, pre and post instrumentation using a high-resolution micro-computed tomography system (SkyScan 1176, Bruker, Belgium) at the Indian Institute of Technology, Hyderabad. Scanning parameters included 80 kV, 200 µA, a 0.5-mm aluminium- copper filter, 360°C rotation with 0.5°C angular steps, a 504-ms exposure time and a voxel size of 16.45 µm. Reconstructed images were processed using NRecon (v1.7.4.2), with quantitative assessment conducted in CTAn and three-dimensional rendering in CTVol. Corrections for flat-field, geometric distortion and ring artifacts were applied. Root canal preparation was performed using three different instrumentation systems. In Group I, canals were prepared using the HyFlex EDM system in the following sequence: (18/.11), (15/.03) and (30/.04) operated at 300 rpm and 1.8 Ncm. #10 K-file was used for recapitulation between instruments. In Group II, canals were instrumented with the TruNatomy system comprising an (20/.08), (17/.02), (20/.04), (26/.04), (36/.03). Instruments were used at 500 rpm and 1.5 Ncm with short (2-3 mm) in-and-out motions. The flutes were cleaned after every two strokes. In Group III, the XP-endo Rise system (FKG Dentaire, Switzerland) was used. The Rise Glider (15/.04) and Rise Shaper (30/.04) were operated at 1000 rpm and 1 Ncm in a continuous, gentle up-and-down motion without pecking. Instruments were allowed to progress passively to working length. The evaluation parameters in this study included canal transportation (CT) and centering ability (CA), both assessed at three standardized levels along the root canal: 3 mm (apical), 6 mm (middle) and 9 mm (coronal). Canal transportation was quantified using Gambill's formula. In the mesiodistal direction, it was calculated as (a_1_ - a_2_) - (b_1_ - b_2_) and in the buccolingual direction, as (c_1_ - c_2_) - (d_1_- d_2_). A value of 0 indicated no transportation, while positive values denoted mesial or buccal transportation and negative values indicated distal or lingual transportation. Centering ability, on the other hand, was computed according to the formula (a_1_ - a_2_)/ (b_1_- b_2_). A value of 1 represented ideal centering of the instrument within the canal, whereas values approaching 0 indicated poor centering. These parameters provide critical insight into the shaping efficiency and anatomical preservation achieved by the tested instrumentation systems. Representative axial micro-CT slices were obtained showing labelled measurements a_1_, b_1_, c_1_, & d_1_ and a_2_, b_2_, c_2_, & d_2_ (Figure 2 - see PDF), Representative pre & post instrumentation micro CT scan images (Figure 3 - see PDF).

## Statistical analysis:

Data were analyzed using SPSS version 23.0 (IBM Corp., Armonk, NY, USA). Normality was assessed with the Shapiro-Wilk test. As transportation values were not normally distributed, non-parametric tests were used: intergroup comparisons (buccolingual and mesiodistal) at apical, middle and cervical thirds were performed with the Kruskal-Wallis test, followed by Dunn's post-hoc test with Bonferroni correction. Intragroup comparisons across root levels within each system employed Friedman's ANOVA with Wilcoxon signed-rank tests for pairwise analysis. Centering ability was evaluated using repeated measures ANOVA for normally distributed data or Friedman's ANOVA where non-parametric; between-group comparisons at each level used one-way ANOVA. A two-tailed P ≤0.05 was considered statistically significant.

## Results:

Analysis revealed no significant differences in buccolingual transportation at the apical region (P = 0.668), middle (P = 0.079), or cervical third (P = 0.552) (Table 1; Figure 4 - see PDF). However, HyFlex EDM consistently showed the least transportation, particularly at the apical third (+0.172 mm), suggesting better canal centering. Mesiodistal canal transportation was greatest with XP Endo Rise in the apical (0.3783 ± 0.46071) and middle thirds (0.4283 ± 0.76324), followed by HyFlex EDM and TruNatomy (Table 2; Figure 4 - see PDF). Although differences were not statistically significant overall (P = 0.806 and 0.079, respectively), the middle third approached significance, indicating a potential trend. At the cervical level, HyFlex EDM demonstrated the greatest transportation value (0.1433); however, the difference among the systems was not statistically significant (P = 0.258). Although the observed differences did not reach statistical significance (P > 0.05), HyFlex EDM showed the best centering ability at the apical and cervical thirds (Table 3; Figure 5 - see PDF). XP-Endo Rise maintained consistent centering, while TruNatomy demonstrated lower centering in the cervical third.

## Discussion:

Micro-computed tomography (micro-CT) has emerged as the reference standard for assessing canal shaping due to its non-destructive, three-dimensional and highly reproducible imaging capabilities [10]. It allows quantitative analysis of canal transportation and centering ability with high accuracy. Nickel-titanium instruments differ in metallurgy and design, influencing their shaping performance. HyFlex EDM, produced by Electrical Discharge Machining, offers superior flexibility and fracture resistance; TruNatomy, with its regressive taper, emphasizes dentin conservation; and XP-Endo Rise, made from MaxWire alloy, adapts to canal curvatures at body temperature. This study assessed the shaping performance of three file systems in curved mesiobuccal canals of extracted mandibular first molars using micro-CT. Shaping ability was assessed based on two parameters: canal transportation, measured in both buccolingual and mesiodistal directions and centering ability, evaluated at three root canal levels. The three nickel-titanium (NiTi) rotary systems demonstrated transportation values within clinically acceptable ranges at all canal levels (<0.3 mm), with no statistically significant differences observed. In the buccolingual direction, all systems exhibited a consistent pattern of buccal deviation apically and lingual deviation coronally, likely due to the interplay between canal curvature and instrument biomechanics. These findings align with previous observations that files tend to straighten towards the outer curve, increasing structural risk in thinner dentin regions near the apex [11]. At the apical third, XP Endo Rise showed the greatest mean transportation, aligning with Shaheen & Elhelbawy *et al.* [12]. TruNatomy exhibited the lowest apical transportation, corroborating findings by Gazzaneo *et al.* [13].

HyFlex EDM's balanced performance supports Rubio *et al.* meta-analysis [14]. Results for the middle and cervical thirds were also comparable among groups. HyFlex EDM showed greater coronal/ cervical and middle shaping consistent with its design and cutting efficiency, whereas TruNatomy tended to maintain lower transportation, in line with prior reports (Kumar *et al.* 2021; Ronquete *et al.* 2022) [15, 16]. XP Endo Rise demonstrated notably low transportation in the cervical third, reflecting the effectiveness of its adaptive core design in preserving canal morphology. This aligns with micro-CT findings that XP-Endo Shaper another Max-Wire rotary system achieves comparable shaping performance to ProTaper Next in oval-shaped canals, with no significant deviations in canal transportation (Velozo *et al.* 2020) [17]. XP Endo Rise showed the highest mean mesiodistal transportation in the apical and middle thirds, though differences were not statistically significant, with a trend in the middle third (P = 0.079). Similar variability has been reported for XP-Endo systems, where their adaptive design influences shaping performance and fatigue resistance (Silva *et al.* 2018; Öztürk *et al.* 2020) [18, 19]. HyFlex EDM exhibited intermediate transportation values, while TruNatomy exhibited the lowest overall canal transportation, particularly in the apical and middle thirds, consistent with CBCT findings reported by Zargar *et al.* (2025) and Kumar *et al.* (2021). Its off-center design and regressive taper likely facilitate conservative shaping, even though such architecture may inadvertently favor lingual wall removal, contributing to variability in transportation outcomes [20]. XP Endo Rise demonstrated strong preservation of canal anatomy in the cervical third likely a reflection of its adaptive-core design. This is supported by CBCT findings showing that XP-Endo Shaper, a system with similar metallurgy and shaping mechanics, achieved better centering and lower transportation compared to ProTaper Universal (Karkehabadi *et al.* 2021) [21]. XP-Endo Rise demonstrated the highest centering ratios across all canal thirds particularly in the cervical third despite higher variability (P > 0.05). This finding is consistent with CBCT-based evidence demonstrating that XP-Endo Shaper exhibited superior centering ability and reduced canal transportation compared with ProTaper Next (Karkehabadi *et al.* 2025) [21], as well as micro-CT reports of superior centering compared to other systems (Langaliya *et al.* 2023) [22]. These outcomes are consistent with the inherent anatomical compliance conferred by its adaptive core design (Alkahtany *et al.* 2024) [23]. The study's limitations include it *in vitro* design and lack of clinical outcome data. Micro-CT scanning is also resource- intensive. Future studies should integrate CBCT for assessing root canal curvatures and evaluate larger and more varied anatomical samples. AI-assisted micro-CT segmentation may improve data analysis consistency [24]. Overall, the study reinforces the concept of system-specific strengths: Hyflex EDM excels in conservative and consistent shaping of apical and cervical thirds; while TruNatomy maintains a more uniformly conservative preparation throughout the canal; and XP-Endo Rise delivers superior control in the cervical third while exhibiting more aggressive apical shaping. This nuanced understanding informs an evidence-based approach for clinicians to select instruments tailored to canal anatomy and clinical objectives, optimizing the balance between effective debridement and preservation of structural integrity. The findings are also congruent with the contemporary principles of minimally invasive endodontics, emphasizing the preservation of pericervical dentin and prevention of iatrogenic errors that compromise tooth strength and prognosis.

## Conclusion:

All three systems demonstrated comparable shaping performance in mesiobuccal canals of mandibular first molars, with transportation and centering values within clinically acceptable limits. TruNatomy was the least aggressive, preserving canal anatomy most effectively; XP-Endo Rise adapted well buccolingually but showed greater mesiodistal transportation in the apical and middle thirds and HyFlex EDM offered balanced shaping with slightly higher transportation in the cervical third. These results highlight system-specific advantages, though instrument selection should ultimately depend on the clinician's expertise and clinical judgment.
